# Cutaneous Manifestations of Nutritional Deficiencies in the Context of Food Deserts of United States

**DOI:** 10.7759/cureus.29464

**Published:** 2022-09-22

**Authors:** Justin P Canakis, Shane M Swink, Nicholas P Valle, Diana A Rivers, Kristina M Lim, Steven Oberlender, Stephen Purcell, Cynthia Bartus

**Affiliations:** 1 Internal Medicine, George Washington University, Washington DC, USA; 2 Dermatology, Lehigh Valley Health Network, Allentown, USA; 3 Internal Medicine, Meharry Medical College, Nashville, USA; 4 Dermatology, Advanced Dermatology Associates, Allentown, USA

**Keywords:** food desert, public health, socioeconomics, dermatitis, nutritional deficiency

## Abstract

Food deserts exist due to a multitude of factors ranging from socioeconomic status, racial disparities, geography, cost, and healthful food access. Given the vast biological function of vitamins and minerals, the clinical presentation for nutritional deficiencies ranges from benign to life-threatening. Often, the first indicators of underlying nutritional deficiencies are cutaneous manifestations. The first patient case is a 36-year-old female at 25 weeks gestation with a pruritic and painful rash that began in the genital region and spread centrifugally to her legs. The second patient case is a 42-year-old male with a pruritic rash that began at his abdomen and progressed to his thighs. The third patient case is a 48-year-old female with scattered lower extremity ecchymoses in different healing stages and scattered perifollicular erythema with corkscrew hairs. All three patients were found to have nutritional deficiencies and lived in identified food deserts. Deficiencies of zinc, vitamin A, thiamine, pyridoxine, and vitamin C and their subsequent cutaneous manifestations have scarce documentation within food deserts. These cases provide further insight into nutritional deficiencies and offer an opportunity for providers to identify patients at risk. To promote wellness, patients suffering from food insecurity must be identified efficiently and connected with essential resources.

## Introduction

In the United States, certain populations are at increased risk of nutritional deficiencies due to a combination of genetic and environmental factors. Given the vast biological function of vitamins and minerals, the spectrum of clinical presentation for nutritional deficiencies ranges from benign to life-threatening. Cutaneous manifestations can be the first indicators of underlying nutritional deficiencies. Early recognition of nutritional deficiency dermatoses can assist physicians in diagnosing and treating underlying and associated systemic diseases. It is important to consider the multitude of risk factors that contribute to vitamin and mineral deficiencies, including the patient’s age, sex, underlying medical conditions, drugs and medications, socioeconomic status (SES), and residence in a food desert. Food deserts are identified as low-income, populated areas where residents lack access to affordable, healthy foods [1]. Public health efforts have mapped out the relationship between income and access to the nearest supermarket and demonstrated the prevalence of food deserts throughout the United States. This case series aims to highlight the association of food deserts and nutritional dermatoses.

## Case presentation

Case one

**Figure 1:**
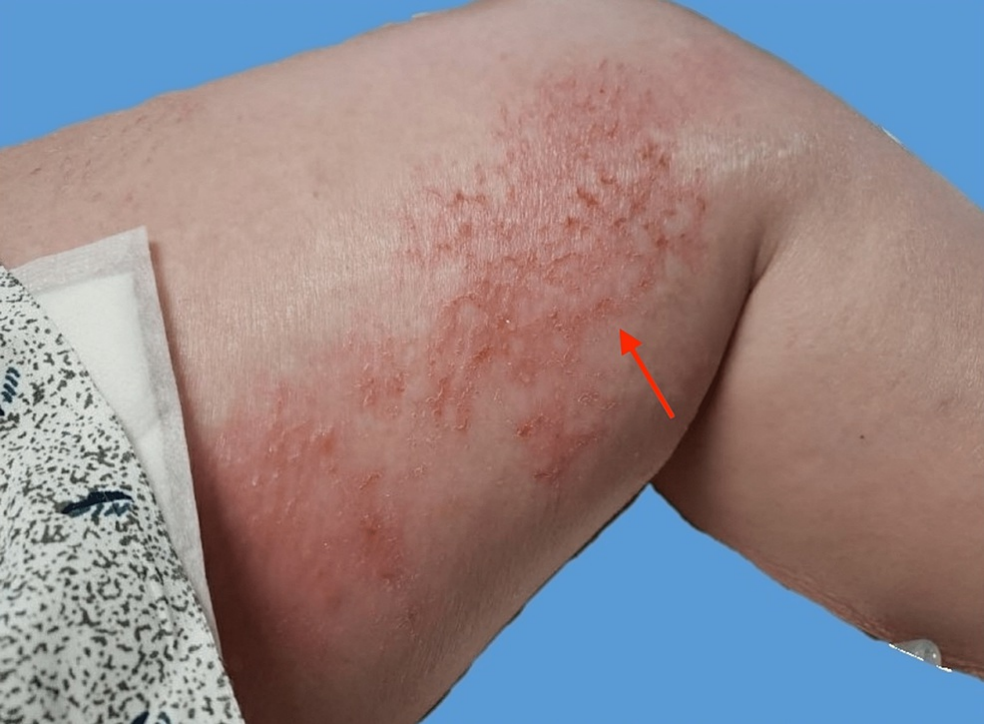
Erythematous, scaly plaques on the left medial thigh with edema and serous drainage

**Figure 2:**
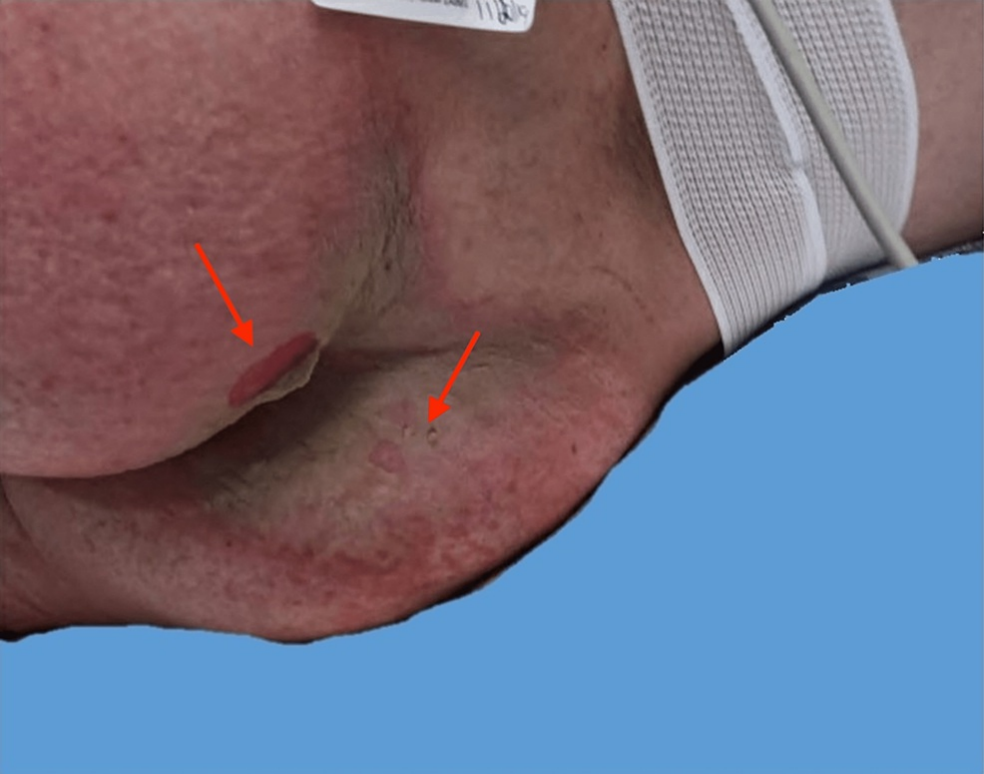
Gluteal cleft with desquamation over an erythematous patch

A 36-year-old female at 25 weeks gestation, with a past medical history of polyarticular juvenile rheumatoid arthritis, bipolar disorder, and depression, presented with a five-day history of a pruritic and painful rash that began in the genital region and spread centrifugally to her legs. She was admitted to the hospital two weeks prior with edema and proteinuria secondary to poor nutritional status in the setting of pregnancy. Social history revealed limited mobility secondary to arthritis and poor access to financial resources. A physical exam revealed erythematous, scaly plaques on the medial thighs, legs, abdomen, and posterior ankles with serous drainage (Figure 1). Examination of the gluteal cleft demonstrated desquamation over an erythematous patch (Figure 2). Additional findings included generalized xerosis and an edematous, erythematous vulva.

**Figure 3:**
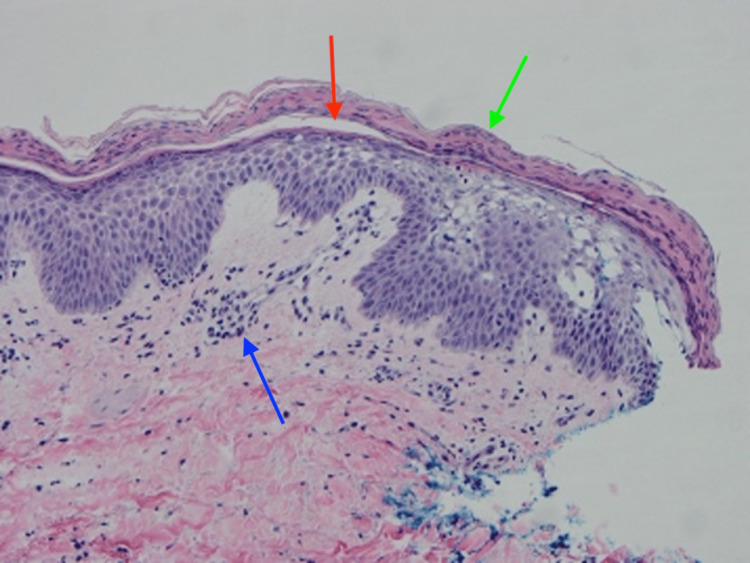
H&E 20x) Right anterior proximal thigh biopsy shows patchy vacuolation and pallor of the upper epidermis, overlying parakeratosis (green arrow), papillary dermal and dermal edema (red arrow) with superficial and deep, predominantly perivascular lymphohistiocytic inflammation (blue arrow)

Laboratory data was significant for zinc 24.8 ug/dL (60-120), vitamin A retinol free <0.06 mg/L (0.3-1.2), vitamin B1/thiamine <2 mmol/L (4-15), and vitamin B6/pyridoxine 6.7 mmol/L (20-150). An echocardiogram did not reveal evidence of cardiac failure. Skin biopsy of the right anterior proximal thigh demonstrated patchy vacuolation and pallor of the upper epidermis, overlying foci of parakeratosis, and papillary and dermal edema with superficial and deep, predominantly perivascular, lymphohistiocytic inflammation (Figure 3). Periodic acid-Schiff-diastase histochemical study was negative for pathogenic organisms.

The patient was treated with triamcinolone 0.1% cream, vitamin B6, zinc sulfate, and prenatal vitamins with improvement of her symptoms within three months. 

Case two

**Figure 4:**
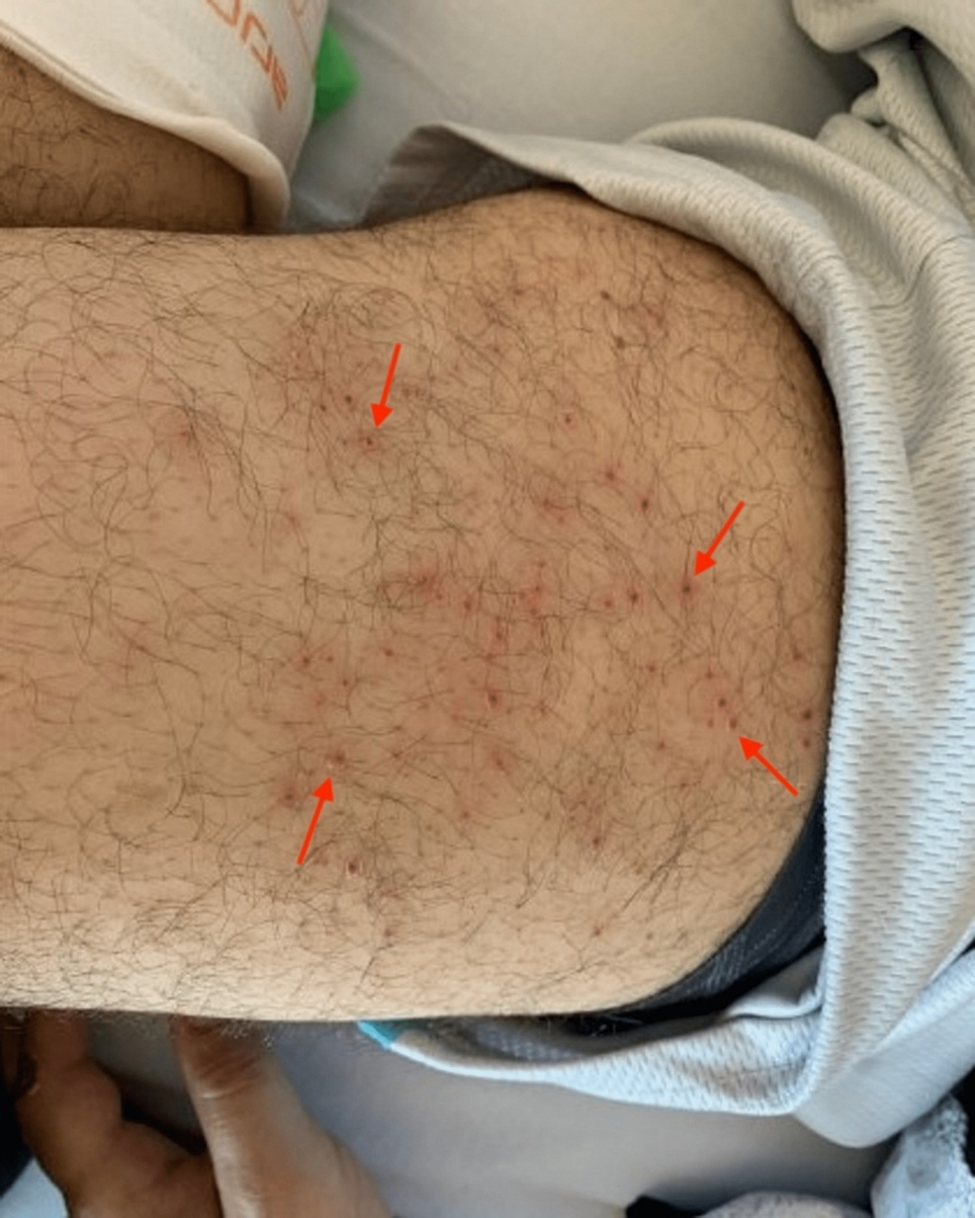
Prominent perifollicular erythema and hyperkeratotic papules of the left anterior thigh

A 42-year-old male with a past medical history of chronic hepatitis C, anxiety, depression, asthma, tobacco use, and intravenous drug abuse was admitted to the hospital with concerns for endocarditis and a rash. The patient reported a one-week duration of subjective fevers, chills, shortness of breath, and pruritic rash. The rash began on his abdomen and progressed to his thighs. Associated symptoms included decreased appetite, nausea, vomiting, painful arches with walking, and easy bruising. He denied gingival bleeding. He reported that his diet consisted of little to no produce due to lack of access. Physical exam of the abdomen revealed ecchymosis on the bilateral lower abdominal quadrants, pink folliculocentric and/or hyperkeratotic papules, and scattered pustules. Examination of the anterior thighs displayed prominent perifollicular erythema and hyperkeratotic papules (Figure 4). Other pertinent findings included a splinter hemorrhage on the left third digit, no gingival bruising, and no evidence of petechiae or corkscrew hairs.

Laboratory analysis was notable for urine drug screen positive for cocaine, heroin, and benzodiazepines and a non-fasting vitamin C level of 0.147 mg/dL (0.4-2). The patient was started on vitamin C supplementation with improvement of his symptoms within two weeks. 

Case three

**Figure 5:**
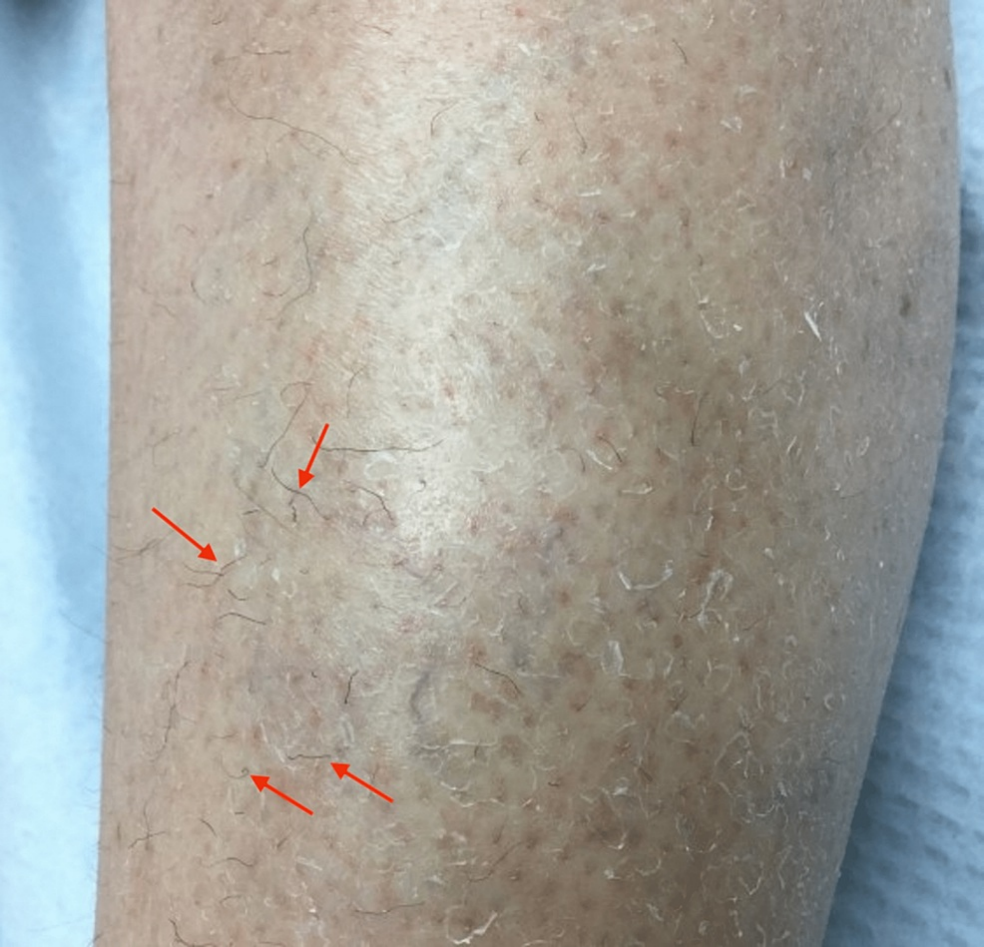
Corkscrew hairs and perifollicular erythema of the right lower extremity

A 48-year-old female with a past medical history of esophagitis, seropositive rheumatoid arthritis, fibromyalgia, depression, anxiety, tobacco use, ambulatory dysfunction, folate deficiency, and vitamin D deficiency presented for evaluation of lower extremity bruising. The patient reported intermittent bruising for many weeks. Associated symptoms included muscle pain and weakness that required recent hospitalization and perifollicular redness, and corkscrew-shaped hair. Social history revealed that she did not consume produce due to a lack of access. Physical exam of the bilateral lower extremities was significant for scattered yellow-purple ecchymoses in different healing stages that were tender to palpation, as well as scattered perifollicular erythema with corkscrew hairs (Figure 5).

At one-month follow-up, she reported mild improvement in ecchymoses and ambulatory dysfunction after introducing large amounts of baby food rich in greens to her diet. Physical exam demonstrated decreased corkscrew hairs on her lower extremities bilaterally, no tenderness to palpation, and a resolution of prior ecchymoses. Plasma vitamin C levels were ordered and revealed a level of 0.09 mg/dL (0.4-2).

She was prescribed vitamin C supplementation 500 mg twice daily for 30 days, then maintenance of 500 mg daily. At the eight-month follow-up, her vitamin C level was 0.8 mg/dL (0.4-2), and a physical exam revealed no ecchymoses and very few corkscrew hairs. 

A summary of all three cases is shown in Table 1.

**Table 1:** Summary of cases

	Case 1	Case 2	Case 3
Past medical history	25 weeks gestation, polyarticular juvenile rheumatoid arthritis, bipolar disorder, and depression	Chronic hepatitis C, anxiety, depression, asthma, tobacco use, and intravenous drug abuse	Esophagitis, seropositive rheumatoid arthritis, fibromyalgia, depression, anxiety, tobacco use, folate deficiency, and vitamin D deficiency
Social history	Reported lack of access to healthy foods	Reported lack of access to healthy foods	Reported lack of access to healthy foods
Presentation	Pruritic and painful rash that began in the genital region and spread centrifugally to her legs	Rash on the abdomen that progressed to his thighs	Intermittent, lower extremity bruising and muscle weakness
Physical exam	Erythematous, scaly plaques on the medial thighs, legs, abdomen, and posterior ankles with serous drainage. Gluteal cleft with desquamation over an erythematous patch. Generalized xerosis and an edematous, erythematous vulva.	Ecchymosis on the bilateral lower abdominal quadrants, pink folliculocentric and/or hyperkeratotic papules, and scattered pustules. Perifollicular erythema and hyperkeratotic papules on anterior thighs. Splinter hemorrhage on the left third digit	Bilateral lower extremities showing scattered ecchymoses in different healing stages that were tender to palpation, as well as scattered perifollicular erythema with corkscrew hairs
Labs	Zinc 24.8 ug/dL (60-120); Vitamin A retinol free <0.06 mg/L (0.3-1.2); Vitamin B1 <2 mmol/L (4-15); Vitamin B6 6.7 mmol/L (20-150)	Vitamin C 0.147 mg/dL (0.4-2)	Vitamin C 0.09 mg/dL (0.4-2)
Treatment	Triamcinolone 0.1% cream, vitamin B6, zinc sulfate, and prenatal vitamins	Vitamin C	Vitamin C

## Discussion

Food deserts exist due to a multitude of factors ranging from socioeconomic status, racial disparity, geography, cost, and accessibility to healthful foods [2]. Supermarkets with healthful options are less prevalent in regions of lower SES, demonstrated by low-income neighborhoods having four times as many non-chain grocery stores and half as many chain supermarkets compared to more affluent neighborhoods [2]. The void of healthful options in low-income urban communities is filled by small non-chain, grocery stores that do not have the capital to buy fresh produce, instead utilizing the cost-effective option of stocking non-perishable packaged foods with a longer shelf-life. This is further demonstrated by racial disparity when comparing predominantly white communities to predominantly black communities, where lower proportions of stores carry fresh produce. Additionally, the cost of fresh food in food deserts, both urban and rural, is higher compared to non-food deserts, further contributing to difficulty accessing good-quality, nutritious food in those food deserts [2]. Food deserts contribute to a unique public health crisis: the malnutrition faced by Americans is often not due to a lack of calories, but rather the overconsumption of calorically dense foods with limited nutritional value. The lack of access to nutritiously rich foods in food deserts is one of the factors that may also contribute to obesity, diabetes, cardiovascular disease, and nutritional deficiencies [3].

Living in a food desert exacerbates the risk of nutritional deficiency in at-risk populations with other comorbidities. In all our cases, the patients had at least one risk factor for nutritional deficiency, including pregnancy, liver disease, drug abuse, and/or a history of vitamin deficiencies. Interestingly, one commonality of our reported patients is that they each live in a different food desert, identified by zip code, according to the United States Department of Agriculture Economic Research Service. All three patients live in neighborhoods classified as low-income and have low access to food [4]. Therefore, checking patients’ zip codes for food desert status can serve as a unique tool for physicians to help assess patients’ risk for developing malnutrition and associated diseases.

There is limited data or research available to date on vitamin and mineral deficiencies in food deserts. Cases of zinc, vitamin A, thiamine, pyridoxine, and vitamin C deficiencies and their subsequent cutaneous manifestations have scarce documentation in food deserts. Nearly one-third of the United States population is at risk of deficiency in at least one vitamin; thus, it is important to understand the presentation of these deficiencies [2]. Relevant to the dermatologist, deficiencies of vitamins and minerals have multiple cutaneous manifestations and other significant complications critical to recognize (Table 2) [5-9]. Regarding minerals, clinicians should be cognizant of iron, zinc, and selenium deficiencies and their respective manifestations. The most common documented mineral deficiency is iron. The odds of having iron deficiency anemia in adolescents and pregnant women with food insecurity, defined as the state of being without reliable access to sufficient quantities of affordable, nutritious food (e.g., living in a food desert), is almost three times higher than those with food security [2, 3]. In the United States, a higher prevalence of low zinc intake was found among older adults (≥60 years) of food-insufficient families versus food-sufficient families [6].

**Table 2:** Overview of Vitamin and mineral biological function, cutaneous manifestations of deficiencies, and associated significant complications of deficiencies NADPH - Nicotinamide adenine dinucleotide phosphate, FAD - flavin adenine dinucleotide, FMN - flavin mononucleotide, NAD - nicotinamide adenine dinucleotide

Vitamin/ mineral	Biological function	Cutaneous manifestations of deficiency	Significant complications of deficiency
Iron	Essential component of hemoglobin	Pallor; dry or rough skin; atrophic glossitis with loss of tongue papillae; angular cheilitis; koilonychia; alopecia; chlorosis	Iron deficiency anemia
Selenium	Antioxidant defense; thyroid hormone production	Hypopigmentation of nail beds	Skeletal muscle dysfunction; cardiomyopathy; mood disorders; impaired immune function
Vitamin A (Retinol)	Protein synthesis in the eye; enhances immune system and keratinocyte function	Xerosis cutis; acneiform lesions; follicular keratosis	Nyctalopia; keratomalacia; immunosuppression
Vitamin B1 (Thiamine)	Essential cofactor involved in NADPH synthesis, macromolecule aggregation, and neural function	Wet beriberi presents with edematous skin with subsequent breakdown and glossitis	Wernicke-Korsakoff syndrome; dry beriberi; wet beriberi
Vitamin B2 (Riboflavin)	Essential cofactor in redox reactions, FAD and FMN are derivatives	Cheilosis; angular stomatitis; keratitis; seborrheic dermatitis; scrotal dermatitis	
Vitamin B3 (Niacin)	Essential cofactor in redox reactions, NAD is a derivative	Erythema Hyperpigmentation of sun-exposed skin; dermatitis with a C3/C4 dermatome; circumferential broad collar rash; seborrheic dermatitis; scrotal dermatitis; glossitis	Pellagra
Vitamin B6 (Pyridoxine)	Cofactor for more than 140 enzymes	Eczematous or seborrheic dermatitis; scrotal dermatitis; angular cheilitis; stomatitis; glossitis; intertrigo	Convulsions; peripheral neuropathy; sideroblastic anemia
Vitamin B7 (Biotin)	Cofactor for carboxylation enzymes	Dermatitis around the eyes, nose, and mouth; alopecia	Hallucinations
Vitamin B9 (Folate)	Essential for methylation reactions and DNA/RNA synthesis	Glossitis	Megaloblastic anemia
Vitamin B12 (Cobalamin)	Cofactor for methionine synthase Important for DNA synthesis	Skin hyperpigmentation; vitiligo; angular stomatitis; atopic dermatitis; acne; hair and nail changes	Megaloblastic anemia; subacute combined degeneration
Vitamin C (Ascorbic acid)	Essential cofactor for hydroxylation of proline and lysine in collagen synthesis; Necessary for dopamine B-hydroxylase; facilitates iron absorption	Swollen gums; ecchymosis; petechiae; poor wound healing; perifollicular and subperiosteal hemorrhages; corkscrew hair	Scurvy; weakened immune response
Vitamin D	Aids in intestinal absorption of calcium and phosphate; bone mineralization and bone resorption	Increased risk of psoriasis and atopic dermatitis	Rickets; osteomalacia
Vitamin K	Necessary for the maturation of clotting factors II, VII, IX, X, protein C, and protein S	Easy bruisability; mucosal bleeding; splinter hemorrhages	Hemorrhage
Zinc	Essential trace element required for metalloproteinases and transcription factors to function in wound repair, immune responses, and reproduction; antioxidant properties against UV radiation damage	Periorificial, acral, and anogenital dermatitis; alopecia; nail dystrophy; stomatitis; angular cheilitis	Acrodermatitis enteropathica

Fat-soluble vitamin deficiencies are common in the United States and are often associated with malabsorption disorders such as pancreatitis. Table 2 explores the manifestations of vitamin A, D, and K deficiencies. It has been demonstrated that residents of food-insufficient households have lower serum concentrations of vitamin A compared to food-sufficient households [6].

Vitamin C is an essential cofactor for hydroxylation of proline and lysine in collagen synthesis, necessary for dopamine beta-hydroxylase, and facilitates iron absorption [7]. Six percent of adults aged 20-59 are considered vitamin C deficient in the United States, and ten percent have low serum vitamin C concentrations [6]. Additionally, the prevalence of vitamin C deficiency in the United States decreases with increasing SES [10].

Countries around the world face similar micronutrient intake inadequacies that are increasingly being documented. A noteworthy study from rural Bangladesh reported limited diversity and very low intake of adequate nutrition, especially with calcium, niacin, iron, riboflavin, folate, vitamin B-6, and vitamin B-12 [11]. Interestingly, another study in Hong Kong reported more food swamps, which are areas with an abundance of unhealthy foods, rather than food deserts [12].

## Conclusions

While often assumed that nutritional deficiencies are limited to developing countries, their existence in food deserts is evident across the United States. It is imperative to recognize the presence of food deserts with resultant food insecurity and its role in health and disease. Patients suffering from food insecurity must be identified and connected with resources and multidisciplinary teams to promote wellness. Dermatologists play a critical role as the first clinicians to identify visual manifestations of nutritional deficiency.
